# Insect Antimicrobial Peptides as Guardians of Immunity and Beyond: A Review

**DOI:** 10.3390/ijms25073835

**Published:** 2024-03-29

**Authors:** Lizhen Zhou, Guanliang Meng, Ling Zhu, Li Ma, Kangkang Chen

**Affiliations:** 1 Department of Plant Protection, College of Plant Protection, Yangzhou University, Yangzhou 225009, China; lizhenzhou@nwafu.edu.cn; 2 Department of Entomology, College of Plant Protection, Northwest A&F University, Yangling 712100, China; 3 Zoological Research Museum Alexander Koenig, Leibniz Institute for the Analysis of Biodiversity Change, 53113 Bonn, Germany; g.meng@leibniz-lib.de; 4 School of Food Science and Technology, Jiangnan University, Wuxi 214122, China; zhuling@jiangnan.edu.cn; 5 College of Plant Protection, Shanxi Agricultural University, Taigu 030810, China

**Keywords:** antimicrobial agents, antimicrobial peptide evolution, antimicrobial peptide regulation, insect

## Abstract

Antimicrobial peptides (AMPs), as immune effectors synthesized by a variety of organisms, not only constitute a robust defense mechanism against a broad spectrum of pathogens in the host but also show promising applications as effective antimicrobial agents. Notably, insects are significant reservoirs of natural AMPs. However, the complex array of variations in types, quantities, antimicrobial activities, and production pathways of AMPs, as well as evolution of AMPs across insect species, presents a significant challenge for immunity system understanding and AMP applications. This review covers insect AMP discoveries, classification, common properties, and mechanisms of action. Additionally, the types, quantities, and activities of immune-related AMPs in each model insect are also summarized. We conducted the first comprehensive investigation into the diversity, distribution, and evolution of 20 types of AMPs in model insects, employing phylogenetic analysis to describe their evolutionary relationships and shed light on conserved and distinctive AMP families. Furthermore, we summarize the regulatory pathways of AMP production through classical signaling pathways and additional pathways associated with Nitric Oxide, insulin-like signaling, and hormones. This review advances our understanding of AMPs as guardians in insect immunity systems and unlocks a gateway to insect AMP resources, facilitating the use of AMPs to address food safety concerns.

## 1. Introduction

Vertebrates are armed with both innate and adaptive immunities, but insects rely solely on innate immunity to navigate their complex environments with microbes, such as bacteria, fungi, and viruses [1,2]. AMPs, also known as host defense peptides, are crucial and evolutionarily conserved components of the innate immune responses [3,4,5]. They are composed of a diverse group of naturally occurring molecules found in various organisms, including humans, animals, plants, insects, and microorganisms [6,7,8,9,10,11].

AMPs work by acting as the initial defense against a diverse array of pathogens such as bacteria, fungi, viruses, and certain parasites [12]. In comparison to antibiotics, AMPs exhibit a wide-ranging capacity to combat bacteria, fungi, viruses, and even cancer cells [13]. Notably, they possess the ability to kill antibiotic-resistant pathogens, making them a promising candidate for clinical applications [14]. The discovery of AMPs across diverse insect species has significantly advanced our understanding of their functions.

The history of AMPs can be traced back to the identification of gramicidins in 1939. Gramicidins belong to a class of naturally occurring peptide antibiotics produced by specific strains of soil bacteria, *Bacillus brevis* and *Bacillus aneurinolyticus*, known for their activity against Gram-positive bacteria [15,16]. Subsequently, the first plant AMP, known as purothionin, was isolated from wheat (*Triticum aestivum*) endosperm in 1942. As a member of the thionin family of AMPs, purothionin is a cationic peptide with antimicrobial properties capable of targeting and eradicating various microorganisms, including bacteria and fungi [17]. In 1962, bombinin, one of the earliest animal AMPs, was discovered in the orange speckled frog [18]. Bombinin is a defense effector against pathogens, playing a crucial role in protecting frogs from bacterial, viral, and fungal infections. In 1980, the first insect AMPs, cecropins, were identified in the pupae of *Hyalophora cecropia* (Insecta: Lepidoptera), representing a significant milestone as the first major group of α-helical AMPs [19]. Cecropins were notably found in the hemolymph and other immune tissues of insects [20]. The number of known AMPs now exceeds 3000 (detailed in the Antimicrobial Peptide Database, http://aps.unmc.edu/AP, accessed on 20 December 2023), suggesting their versatile functionalities beyond antimicrobial activity and encompassing roles in wound healing, inflammation modulation, and endotoxin neutralization. Overall, the study of AMPs has offered invaluable insights into the ancient origins of defense mechanisms and their evolution, inspiring potential applications as alternatives to conventional antibiotics and fueling biomedical research for novel therapeutic purposes.

Some reviews have summarized the AMPs from the model insect *Drosophila* (Insecta: Diptera), which have mainly focused on the classification, activity, and regulation of AMPs [21,22]. In this review, we summarize the current knowledge and recent advances on AMPs from various model insects, highlight the regulatory pathways and evolution of insect AMPs, and present a perspective on the potential applications of insect AMPs.

## 2. Structures, Antimicrobial Activities, and Common Properties of Insect AMPs

### 2.1. Structures and Antimicrobial Activities of Insect AMPs

Different AMPs may exhibit diverse activities against bacteria, fungi, or viruses based on their structures. Understanding how AMPs function in defending against microbial pathogens relies on uncovering the relationship between their structure and activity. The quantities and types of AMPs in insects vary significantly between species (Table 1). Insect AMPs are typically categorized into four groups based on their secondary structures: α-helical AMPs, β-sheet cysteine-rich AMPs, proline-rich AMPs, and glycine-rich AMPs [23,24,25].

#### 2.1.1. α-Helical Insect AMPs

Insect α-helical AMPs (e.g., cecropin and moricin) contain N-terminal amphiphilic α-helixes and C-terminal hydrophobic α-helixes [33,34]. They belong to secreted proteins, and mature active AMPs are produced following the removal of signal peptides [25]. The most abundant α-helical peptides are cecropin and cecropin-like peptides, including ceratotoxin, enbacin, hyphancin, sarcotoxins, spodopsin, and stomoxyn [35,36]. Cecropins have a broad range of activities to destroy Gram-positive and Gram-negative bacteria and fungi [37,38,39]. Moricins are specifically found in lepidopteran insects and share a similar secondary structure to cecropin, but cecropin has a hinge region to link the N- and C-terminal α-helixes, which is absent in moricin [25]. Likewise, moricins can fight against Gram-positive/negative bacteria and fungi [26,38,39].

#### 2.1.2. β-Sheet Cysteine-Rich Insect AMPs

In the β-sheet cysteine-rich subclass, AMPs (e.g., defensin and drosomycin) have conserved cysteine residues to form disulfide bonds. These AMPs are composed of α-helixes and β-sheets that are stabilized by disulfide bridges [40]. Defensins are the most representative AMPs in this class. They are widely reported in insect orders, such as Coleoptera, Diptera, Hymenoptera, and Lepidoptera [41,42,43,44]. They have strong activity for killing Gram-positive and Gram-negative bacteria, and some of them, such as *Bombyx mori* (Insecta: Lepidoptera) defensin, *Galleria mellonella* (Insecta: Lepidoptera) gallerimycin, and *Apis mellifera* (Insecta: Hymenoptera) royalisin, also defend against fungi [45,46,47,48,49]. Drosomycin and defensin have similar structures, whereas their sequences differ except for the presence of cysteine residues [40]. As known, drosomycins are antifungal peptides, but they also function in killing Gram-negative bacteria [45,50,51].

#### 2.1.3. Proline-Rich Insect AMPs

The proline-rich subclass AMPs are characterized by the presence of multiple proline residues. Some proline-rich peptides, such as lebocin, drosocin, and apidaecin, undergo O-glycosylation as a post-translational modification, which is essential for maximizing their activity [52,53]. However, proline-rich AMP abaecin from *A. mellifera* is not O-glycosylated [54]. Lebocins, sharing 41% sequence identity to *A. mellifera* abaecin, are proline-rich glycopeptides reported in lepidopteran insects. They are generated by proteolytic activation of their precursor proteins, and activated lebocins can broadly defend against Gram-positive/negative bacteria and fungi in lepidopteran insects [55,56,57]. Drosocin is a typical short-sized proline-rich glycopeptide found in *D. melanogaster*, and its primary antimicrobial activity is targeted against Gram-negative bacteria [58,59]. Apidaecins are the major AMPs in *A. mellifera* humoral immunity. They share significant sequence homology with drosocin and are effective in defending against Gram-negative bacteria [60].

#### 2.1.4. Glycine-Rich Insect AMPs

Glycine-rich AMPs have been identified in various insect orders, including Coleoptera, Diptera, Hemiptera, Hymenoptera, and Lepidoptera. The common AMPs in this subclass are attacin, coleoptericin, tenecin, diptericin, hemiptericin, hymenoptaecin, and gloverin [12,42,61,62,63]. Attacins are large glycine-rich peptides with a signal peptide, a pro-peptide region, an attacin domain, and two glycine-rich domains [64,65]. Attacins have six isoforms that can be divided into two categories: basic attacins (A–D) and acidic attacins (E and F) [66]. In *D. melanogaster*, attacins A-D mainly kill Gram-negative bacteria. However, attacins also fight against Gram-positive bacteria and fungi in lepidopteran insects [67,68,69]. Gloverins are another group of large glycine-rich peptides identified exclusively in Lepidoptera. Although sharing a high sequence identity among lepidopteran species [70,71], gloverins show varying activities against microbes. Gloverins from *B. mori* mainly fight against Gram-negative bacteria like *Escherichia coli* [72,73], whereas *Spodoptera exigua* (Insecta: Lepidoptera) and *M. sexta* (Insecta: Lepidoptera) gloverins show activity against Gram-positive bacteria but no activity against *E. coli* [70,74]. Diptericins are glycine-rich AMPs that are mainly found in Dipteran, such as *D. melanogaster*, *Sarcophaga peregrina* (Insecta: Diptera), *Mayetiola destructor* (Insecta: Diptera), and *Phormia terranovae* (Insecta: Diptera), and have activities against Gram-negative bacteria [45,75,76,77,78].

### 2.2. Common Properties of Insect AMPs

Although AMPs exhibit diversity in both structure and antimicrobial activity, they share several properties, including heat stability, positive charge, hydrophobicity, and amphipathicity. Some of these properties are necessary for AMP activity and selectivity. Firstly, most insect AMPs have a net positive charge. This cationic nature enables them to interact with negatively charged components of microbial membranes through electrostatic attraction, which is the basis for the sequent aggregation of AMPs on the microbial surface and reaching the concentration threshold for membrane rupture. Studies also show that appropriately increased positive charge enhances the antimicrobial activity of AMPs [79,80]. Secondly, hydrophobicity is a critical property that determines the extent of insertion of AMPs into the microbial membrane phospholipid bilayer. AMPs exhibit hydrophobicity because they contain up to 50% hydrophobic residues, optimal hydrophobicity is necessary for their antimicrobial activity, and higher or lower hydrophobicity results in AMP inactivation [81]. The last characteristic is the amphiphilicity of AMPs. This refers to AMPs having a positively charged hydrophilic region that binds to negatively charged components and a hydrophobic region that binds to lipids. Amphiphilicity is the key feature for AMPs to disrupt the structure of microbial membranes [82]. These characteristics, such as charge, also affect the selectivity of AMPs; for example, cationic AMPs present stronger attraction to the negatively charged bacteria rather than “self”-cells, as the membranes of “self”-cells are composed of zwitterionic phospholipids [23]. It is important to note that these properties interdependently affect the activity and selectivity of AMPs, and changes in one parameter always lead to compensatory changes in others [83].

## 3. Evolution of Insect AMPs

**Figure 1:**
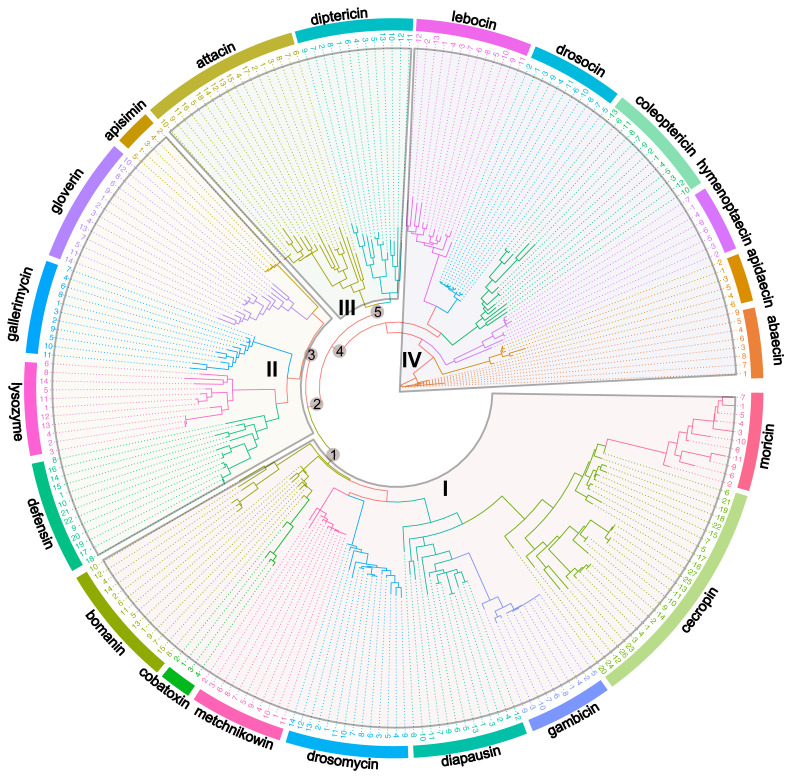
The unrooted Maximum-likelihood tree of different AMP genes. The tree was constructed with IQ-TREE and published sequences, with the optimal evolutionary model (Q.pfam+R4) determined by the ModelFinder algorithm. The sequences are colored by genes (i.e., the names on the outer circle), while the tip labels of the tree are the sequence names. The AMPs are tentatively classified into four groups (I, II, III, and IV). SH-aLRT supports from node 1 to 5 (i.e., the inner node labels near the root) are 71, 93.2, 64, 86.9, 53.9.

To explore the evolutionary history of AMPs across model insects, as well as their diversity and function, we performed the phylogenetic relationships analysis of 20 kinds of AMPs and lysozymes across model insects (Table S1, Supplementary File S1). Based on our phylogenetic analysis, four major groups of AMPs are distinguished (Figure 1). Group I contains eight kinds of AMPs: moricin, cecropin, gambicin, diapausin, drosomycin, metchnikowin, cobatoxin, and bomanin; group II is clustered by lysozymes and four kinds of AMPs: defensin, gallerimycin, gloverin, and apismin; group III is composed of only two kinds of AMPs: attacin and diptericin; group IV includes six kinds of AMPs: lebocin, drosocin, coleoptericin, hymenoptaecin, apidaecin, and abaecin (Figure 1). We found these AMPs are broadly clustered according to their structure and activity. For example, cecropin and moricin (α-helical AMPs), attacin and diptericin (glycine-rich AMPs), and drosocin and lebocin (proline-rich AMPs) cluster together based on their respective structures. Although the secondary structures are not identical, consistent antimicrobial activity (antifungal) may account for the clustering of diapausin, metchnikowin, drosomycin, and bomanin (Figure 1). This indicates that the AMPs within the same major group show close phylogenetic relationships, particularly these AMPs clustered together within smaller clusters.

**Figure 2:**
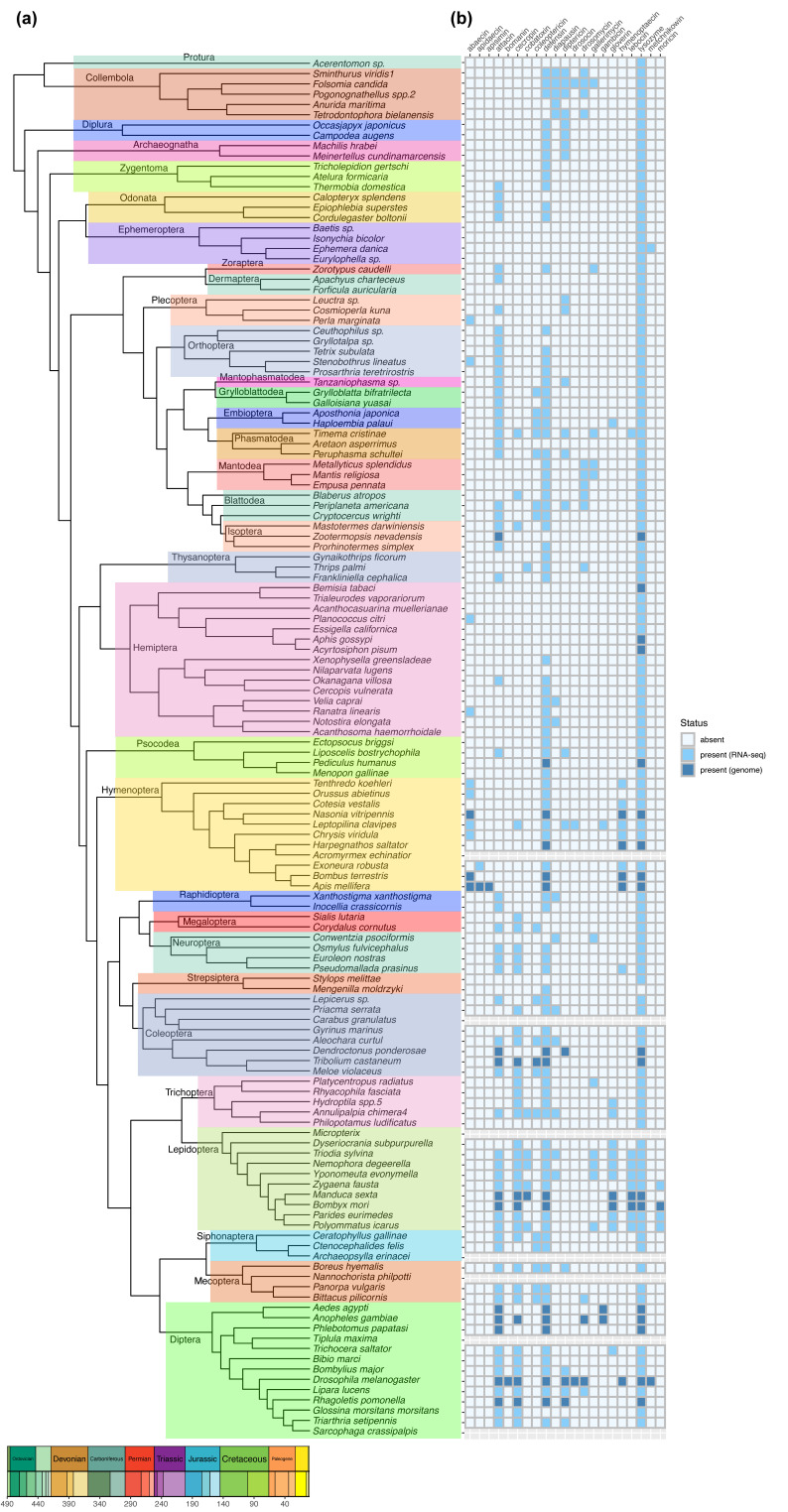
The phylogenetic distribution patterns of AMPs across insect orders. (**a**) The phylogenetic relationships of different insect orders, adapted from Misof et al., 2014 [84]. The chronostratigraphic scale at the bottom shows the divergence times of different insects. (**b**) The distribution pattern of 20 AMPs and lysozyme. Light blue and dark blue, presence of AMPs; white, absence of AMPs; gray background, data not available. Light blue indicates that the determination of AMPs was based on RNA-seq data, while dark blue indicates the determination of AMPs was based on the protein dataset of the corresponding genomes.

The phylogenetic relationships of insect orders are well documented [84] (Figure 2a), while our understanding of the evolutionary patterns of AMPs and lysozymes in insects remains limited. To address this knowledge gap, the distribution patterns of genes responsible for encoding AMPs and lysozymes were explored within the context of Hexapoda evolution. The homologous genes of lysozymes and the 20 kinds of AMPs were identified based on the genomic and transcriptome sequence data (Table S2). The detailed methods are described in Supplementary File S1.

Lysozymes can be encoded in all insects, with the exception of *Mengenilla moldrzyki* (Insecta: Strepsiptera), whereas not all kinds of AMPs can be encoded in insects (Figure 2b). Defensins are found in most insects, with over half of insect species having the ability to encode attacins. The wide distribution may be due to their broad-spectrum microbe-killing activities, which fulfill the essential need of insects to destroy pathogens, allowing defensin and attacin to be largely retained over the course of evolution. In contrast, apidaecin and apisimin are uniquely present in specific bee species (Figure 2b). Bomainins can only be found in *D. melanogaster*, and moricins are present in some lepidopteran insects (Figure 2b). Taken together, some AMPs are highly conserved and widely distributed across different species, while some AMPs are unique to specific species.

In the order of Lepidoptera, insects exhibit the most diverse AMP types (Figure 2); most insects in this order produce a greater number of AMPs than any other taxonomic group [24]. Some insect species have no AMP production in response to immune challenge, exemplified by some Hemiptera insects. Hemiptera are distinctly divided into two groups (Figure 2a). The first group includes *Trialeurodes vaporariorum*, *Bemisia tabaci*, *Acanthocasuarina muellerianae*, *Planococcus citri*, *Essigella californica*, *Acyrtosiphon pisum*, and *Aphis gossypii* (Figure 2a), where most species lack all AMP genes, whereas only one kind of AMP gene (*abaecin*) is found in *P. citri* (Figure 2b). *Acanthosoma haemorrhoidale*, *Notostira elongate*, *Ranatra linearis*, *Velia caprai*, *Xenophysella greensladeae*, *Nilaparvata lugens*, *Cercopis vulnerate*, and *Okanagana villosa* form the second group (Figure 2a). Interestingly, most species in this group can encode one or two kinds of AMPs, at least including defensin, while only *N. lugens* lacks all AMP genes (Figure 2b), which is consistent with our previous study [85]. The absence of all AMPs is also observed in Protura, Ephemeroptera (such as *Baetis* (Insecta: Ephemeroptera), *Isonychia bicolor* (Insecta: Ephemeroptera), and *Eurylophella* (Insecta: Ephemeroptera), and *Strepsiptera* (like *Stylops melittae*) (Figure 2b). Surprisingly, *Philopotamus ludificatus* (Insecta: Trichoptera) lacks all the AMP genes, while the other insects in Trichoptera show a wide variety of AMP types (Figure 2b). The shared occurrence of the absence of all AMPs across different insect orders suggests a possible result of parallel evolution in these insects. However, this could also result from the potential incompleteness of transcriptome and genome datasets or failure in the detection method.

## 4. Action Mechanisms of AMPs

**Figure 3:**
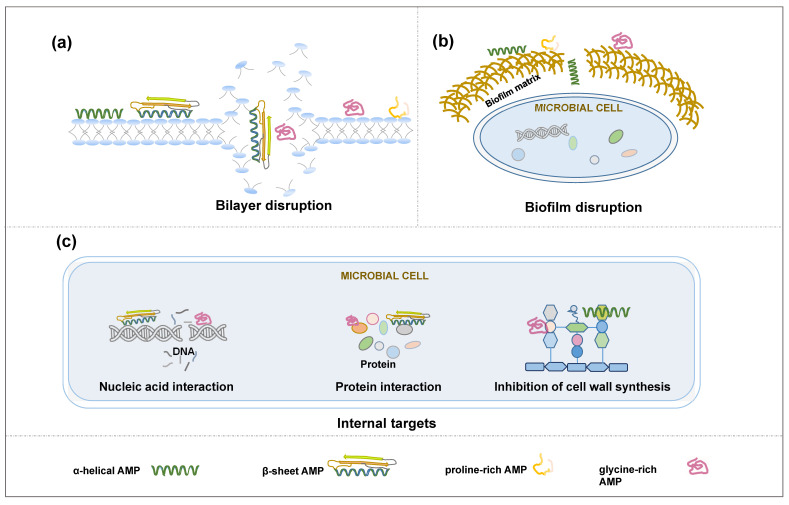
Mechanisms of action of AMPs. These mechanisms are (**a**) bilayer disruption: AMPs insert into the microbial membrane, disrupting membrane integrity; (**b**) biofilm disruption: AMPs penetrate the biofilm matrix, which is produced by microbes, and enter to kill the embedded pathogens; and (**c**) targeting internal components: AMPs target intracellular macromolecules that participate in nucleic acid, protein, and cell wall synthesis to block cell physiological processes.

Bacteria can develop resistance to antibiotics in response to unreasonable antibiotic treatment [86], while AMPs have shown their attractiveness as potential antimicrobial agents [87]. AMPs play a crucial role in the insect’s innate immune system’s defense against pathogens such as bacteria, viruses, fungi, and even some parasites. Here are some mechanisms of how AMPs kill pathogens:

One of the most common mechanisms of action for AMPs is the disruption of the target pathogen’s cell membrane (Figure 3a). Most AMPs have both hydrophobic and hydrophilic regions. They have the capability of inserting themselves into the lipid bilayer of pathogens, forming pores or disrupting the membrane’s integrity [88,89,90]. AMP-17, a novel type of AMP from *Musca domestica* (Insecta: Diptera), destroys 21.7% of the *Candida albicans* (Fungi: Ascomycota) cell wall [91]. This disrupts the pathogenic ability to maintain osmotic balance, leading to cell lysis [92,93]. There are several proposed AMP-mediated disruption models, such as toroidal-pore, barrel-stave, aggregated, and carpet models [94,95,96,97]. Unlike the significant damage to the pathogen’s cell membrane integrity, the other important AMP action is the alteration of the permeability of pathogen cell membranes through a pore-forming transmembrane channel [97,98,99], allowing ions and other essential molecules to leak out and toxic molecules to enter, which, in turn, leads to a disruption in ion balance that can lead to cell death [100,101].

There are some other mechanisms of how AMPs act on pathogens. Some AMPs can penetrate the pathogen’s cell membrane and interfere with essential cellular processes (Figure 3b). These AMPs can interact with pathogen macromolecules, such as key enzymes related to DNA/RNA, protein, and cell wall synthesis, which, in turn, inhibit the growth of pathogens [102]. They may bind ribosomes, DNA, or other vital cellular components, disrupting protein synthesis, DNA replication, and other metabolic processes [103,104,105]. Biofilms are protective structures formed by some bacteria and fungi that can make them resistant to antibiotics [106]. AMPs can disrupt biofilms by penetrating the extracellular matrix and killing the embedded pathogens [107,108,109,110] (Figure 3c).

## 5. Transcriptional Regulation of AMPs

As antimicrobial effectors, insect AMPs are produced in hemocytes, fat bodies, and epithelial cells via two major nuclear factor-κB (NF-κB) pathways during infection: the Toll and the IMD (immune deficiency) pathways [21,111,112,113]. Some AMPs are produced only upon immune stimulation, for example, cecropins. Lysozyme is present at low constitutive levels and significantly enhances transcription in response to immune challenge [27]. Although it has been well described in the model insect *D. melanogaster* that AMPs are transcriptionally induced via the Toll, IMD, and Janus kinase/signal transducer and activator of transcription (JAK-STAT) pathways [113,114], the production and regulation of AMPs in other species remain largely unknown.

### 5.1. Insect AMPs Regulated by Toll Pathway

**Figure 4:**
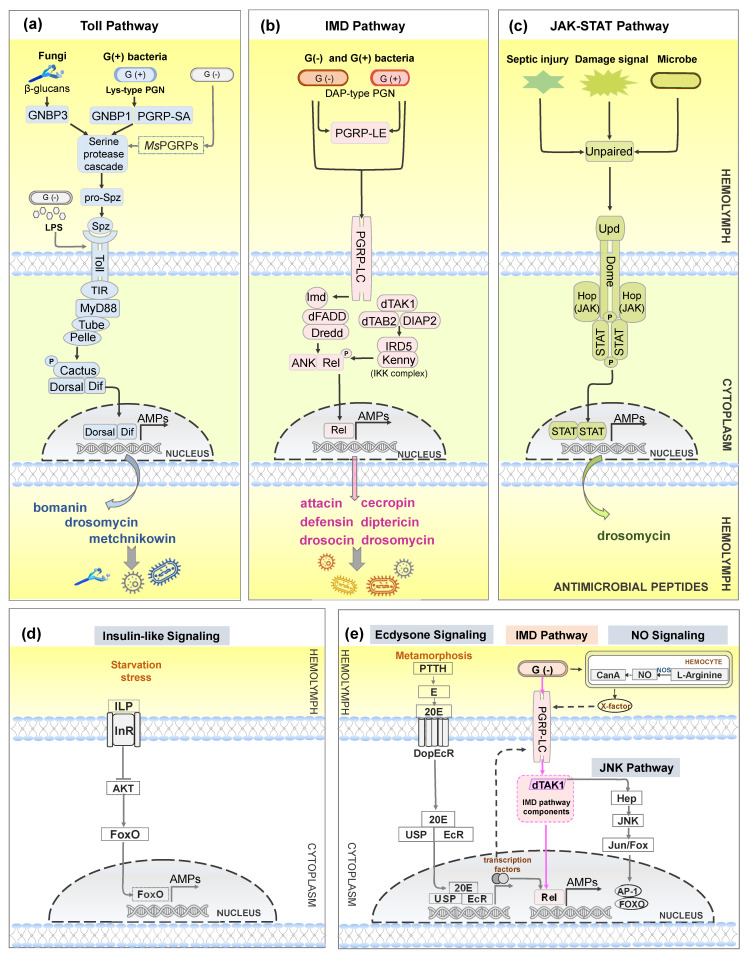
An overview of signal pathways for producing AMPs in insects. The model is mainly based on the immune pathways of *Drosophila*. NF-κB pathways are activated upon binding with bacteria and fungi cell wall components by recognition modules. (**a**) In the Toll pathway, cell surface recognition molecules PGRP-SA, PGRP-SD, and GNBP1 target Lys-type PGN of Gram-positive bacteria, and GNBP3 recognizes β-glucan of yeast and entomopathogenic fungi to activate Toll signaling. Contrary to common belief, *Ms*PGRPs (from *M. sexta*) bind to DAP-type PGN of Gram-negative bacteria to activate the Toll pathway. Toll9 from *B. mori* acts as a pattern recognition receptor and directly binds lipopolysaccharide (LPS) to initiate the Toll pathway without Spätzle binding. Eventually, transcription factor Dorsal and/or Dif translocate into the nucleus to induce the expression of AMP genes (*bomanin*, *drosomycin*, and *metchnikowin*). (**b**) In the IMD pathway, pathogen recognition receptors PGRP-LC and PGRP-LE recognize DAP-type PGN of Gram-negative bacteria and some Gram-positive bacteria. These recognition receptors recruit the IMD adaptor to finally activate the transactivator Rel, and Rel translocates into the nucleus to initiate transcription of specific AMP genes (*attacin*, *cecropin*, *defensin*, *diptericin*, *drosocin*, and *drosomycin*). (**c**) In the JAK-STAT pathway, damage signals/septic injury or pathogens induce Unpaired (Upd) expression, and the pathway is activated through binding of Upd to Dome. STAT, which is phosphorylated by JAK, then separates from Dome, dimerizes and enters into the nucleus to induce AMP transcription (*drosomycin*). Additionally, AMPs can be regulated by other signaling pathways, including insulin-like signaling, ecdysone signaling, NO signaling, and the JNK pathway. In brief, (**d**) starvation stress triggers FOXO activation by insulin-like signaling, and FOXO translocates into the nucleus, initiating the expression of AMPs without microbe challenge. (**e**) Gram-negative bacteria activate NO signaling (NOS oxidizes Arginine to generate NO) in hemocytes, and the released X-factor triggers AMP production by IMD pathways in *Drosophila*. The JNK pathway consists of TAK1, Hep, JNK, Jun/Fox, and transcription factors FOXO and AP-1. Appropriate activation of JNK signaling, which shares a kinase dTAK1 with the IMD pathway, contributes to AMP production. Ecdysone signaling also regulates AMP production by the IMD pathway in *Drosophila*. Prothoracicotropic hormone (PTTH) promotes the synthesis of ecdysone. Active 20E binds to nuclear receptor EcR/USP, initiating the expression of a series of transcription factors. Subsequently, transcription factors up-regulate PGRP-LC expression to activate the IMD pathway or directly initiate AMP expression by the IMD pathway. E: Ecdysone. Dashed arrows indicate that the path is deduced.

The Toll pathway is an evolutionarily conserved signaling cascade and is activated after detection of foreign microbial cell wall components by peptidoglycan recognition proteins (PGRPs). In *D. melanogaster*, PGRP-SA, PGRP-SD, and Gram-negative bacteria-binding protein 1 (GNBP1) recognize the lysine-type peptidoglycan (Lys-type PGN) of Gram-positive bacteria, and GNBP3 involves the sensing of β-glucans of fungi to activate the Toll pathway via the serine protease cascade [115,116]. Previous studies have widely characterized that the Toll pathway responds to invasion by Gram-positive bacteria and fungi. However, a recent finding in *M. sexta* revealed that PGRPs bound to the meso-diaminopimelic acid-peptidoglycans (DAP-type PGNs) of Gram-negative bacteria can also activate Toll signaling [117]. Toll signaling is triggered when extracellular mature cytokine Spätzle binds to the transmembrane Toll receptor. However, Toll9 from *B. mori* can directly bind Gram-negative bacteria-associated ligand lipopolysaccharide (LPS) to initiate the Toll pathway without Spätzle binding [4]. Then, three intracellular proteins, MyD88, Tube adaptor, and Pelle, are recruited to form a heterotrimeric complex that can phosphorylate and degrade Cactus. Activated Dorsal and/or Dif translocate into the nucleus to initiate transcription of AMP genes, such as *bomanin*, *drosomycin*, and *metchnikowin* (Figure 4a).

### 5.2. Insect AMPs Regulated by IMD Pathway

AMPs are also induced from another NF-κB pathway, the IMD pathway. The signaling is activated in the fat body, together with the Toll pathway, during systemic infection. The IMD pathway also produces AMPs in the insect gut to eliminate ingested pathogens, mounting a localized immune response [118,119,120]. Studies in *Drosophila* show that the IMD pathway is triggered by transmembrane PGRP-LC after binding with DAP-type PGN from nearly all Gram-negative bacteria and certain Gram-positive bacteria in systemic immune responses [121,122,123]. Upon binding, PGRP-LC recruits intracellular adaptor protein IMD. The downstream proteins of IMD are *Drosophila* Fas-associated death domain (dFADD) adaptor, Death related ced-3/Nedd2-like caspase (Dredd), TGF-beta activated kinase 1 (TAK1), TAK1-associated binding protein 2 (TAB2), Death-associated inhibitor of apoptosis 2 (DIAP2), IκB kinase (IKK) complex (containing IRD5 and Kenny), and transcription factor Relish. Relish is cleaved into Rel domain and ankyrin-repeat domain (ANK) by Dredd once Relish is phosphorylated by the IKK complex. Further, activated Rel domain translocates into the nucleus to initiate AMP expression, such as *attacin*, *cecropin*, *defensin*, *diptericin*, *drosocin*, and *drosomycin*, to combat pathogens [22,122,124] (Figure 4b).

### 5.3. Insect AMPs Regulated by Intestinal IMD-NF-κB Pathway

AMPs generated from the intestinal IMD-NF-κB pathway have been extensively reported in insects [125,126,127,128]. In *Drosophila*, this local immune response is triggered by cytosolic PGRP-LE after recognition of pathogenic bacteria elicitors [121,123]. AMPs (e.g., diptericin) produced locally in the gut play a critical role in defending against foodborne pathogens [119]. Do these AMPs also kill commensal bacteria along the gut? A study has shown that negative regulators (e.g., PGRP-SC2) of the IMD pathway dampen AMP production and maintain commensal bacterial colony homeostasis in the gut [129]. However, the molecular mechanisms whereby AMPs target only pathogenic bacteria while maintaining symbiotic colony homeostasis are not fully established in *D. melanogaster*. Recently, one study in the oriental fruit fly *Bactrocera dorsalis* (Insecta: Diptera) has revealed this mechanism. In brief, PGRP-LC of *B. dorsalis* positively activates the IMD pathway to generate AMPs in the foregut, filtering pathogenic bacteria from entering the midgut to protect symbiotic bacteria homeostasis. Furthermore, symbiotic bacteria enhance the expression of PGRP-LB and PGRP-SB, which are negative regulators of the IMD pathway, avoiding the threat of AMPs in the *B. dorsalis* midgut. Regional production of AMPs establishes a protective region for symbiotic bacteria [130]. However, some hemipteran insects are deficient in AMPs produced by the IMD pathway due to their lack of crucial compositions such as PGRPs, IMD, dFADD, Dredd, IKK, and Relish [86,131,132]. What is noteworthy is that these insects can survive normally and even exist ubiquitously in the absence of the vital IMD pathway. For example, the aphid, which is a hemipteran insect with an IMD pathway deficiency, can still ravage in nature. The reasons may be as follows: firstly, aphids are insects that adopt the R-strategy to fit the environment, so they invest more energy into reproduction rather than shaping immune resistance under limited resources [133]. Secondly, the phloem sap that aphids feed on is normally sterile, and it largely reduces the risk of oral infection to aphids. Moreover, endosymbiotic bacteria protect aphids against pathogens and parasitoids [86,134,135,136].

### 5.4. Insect AMPs Regulated by JAK-STAT Pathway

JAK-STAT pathway is also a conserved intracellular cascade that modulates the production of AMPs [118,137,138]. This pathway also takes place in the fat body and gut and is elicited upon damage signals such as septic injury, not just pathogen intrusion [46,139,140,141]. The JAK-STAT pathway is initiated through binding with either of the cytokines of Unpaired family (Upd1, 2, and 3) to the receptor Domeless (Dome). This signal recruits the JAK kinase Hopscotch (Hop) to phosphorylate Dome and Stat92E (STAT). Then, dimerized STAT translocates into the nucleus to activate the transcription of target genes such as the AMP gene drosomycin-like peptide [46,137,138,142] (Figure 4c).

We summarize the types and activities of AMPs that are induced by the three conserved pathways mentioned above. The data are collected from some model insects, such as *D. melanogaster*, *M. sexta*, *B. mori*, *Helicoverpa armigera* (Insecta: Lepidoptera), *Tribolium castaneum* (Insecta: Coleoptera), *Anopheles gambiae* (Insecta: Diptera), and *A. mellifera*, based on the available studies (Table 2). Among these, the pathways of AMP production in *Drosophila* are well defined, and most AMPs are induced via the IMD pathway. The IMD pathway tightly controls immune responses in the fat body, hemocytes, and the gut, playing a critical and ancestral role in defending against pathogens [120], whereas the Toll and JAK-STAT pathways have multifunctional roles and are active in developmental processes. Therefore, the sole role of IMD signaling in the immune system allows it to respond more rapidly and readily to invading bacteria [46].

### 5.5. Insect AMPs Regulated by Other Signaling Pathways

Other signaling pathways can also regulate the production of AMPs (shown in Figure 4d,e). Studies show that Nitric Oxide (NO) signaling can trigger AMP expression. However, the mechanisms by which NO regulates AMP production vary across different insects. In *Spodoptera exigua* (Insecta: Lepidoptera), NO signaling regulates AMP production by Toll and IMD pathways together [153]. In *Drosophila*, NO-induced AMP production is dependent only on the IMD pathway [154]. Similarly, a recent study showed that NO also induced AMP expression via the IMD pathway in *Ostrinia furnacalis* (Insecta: Lepidoptera) [155]. Apart from this, appropriate induction of c-Jun N-terminal kinase (JNK) signaling, which branches out from the IMD pathway at dTAK1, is required for the IMD pathway to produce AMPs [156,157].

It is well established that AMP generation is affected by insect endogenous hormones, steroid 20-hydroxyecdysone (20E), and sesquiterpenoid Juvenile hormone (JH) [158,159]. Ecdysone signaling regulates AMP production during insect metamorphosis [160,161,162]. In *Drosophila*, prothoracicotropic hormone (PTTH) regulates the synthesis of 20E precursor ecdysone (Figure 4e). The precursor is then released into the hemolymph and converted to activated form 20E. Then, 20E binds to the membrane receptor DoEcR and enters the cell. Subsequently, 20E binds to nuclear receptor EcR/USP, initiating the expression of a series of transcription factors. Transcription factors can directly initiate AMP expression or activate the IMD pathway by up-regulating PGRP-LC expression [161]. JH suppressed AMP gene expression (e.g., *diptericin*) in *Aedes aegypti* (Insecta: Diptera) [163].

Under starvation stress, AMPs can be induced by insulin-like signaling (IIS). The signaling is initiated upon binding of insulin-like peptide (ILP) to insulin-like receptor (InR). Energy shortage status inhibits IIS signaling and reduces AKT kinase expression. Activated FOXO translocates into the nucleus, inducing the expression of AMPs and anabolic genes [164,165,166]. In addition, AMPs can also be triggered under temperature stress [167]. These pathways differ from the classical AMP signaling pathways, as they can activate the expression of AMPs in the absence of pathogen stimulation.

## 6. Potential Applications of Insect AMPs

To date, AMPs have been found in almost all invertebrates examined, mainly including insects and marine invertebrates. Although some marine invertebrates, such as horseshoe crabs, can produce the antibacterial and antifungal peptides tachyplesin and polyphemusin [82], insects possess distinct advantages as substantial and renewable reservoirs of AMPs. Their short lifecycles, compact size, and ease of cultivation make them ideal candidates for AMP applications. Insect AMPs, with their diverse range and promising application potential, stand as potent microbial agents within the innate immune system. As a result, they have garnered substantial interest throughout the food, agriculture, and pharmaceutical industries.

With growing concerns about food safety, people prefer foods with fewer chemical preservatives and fewer processing procedures. There is a growing trend to use natural preservatives in the food industry. AMPs emerge as alternatives to traditional preservatives, as they exhibit some advantages, such as broad-spectrum bactericidal ability, thermal stability, acid and alkali resistance, and can be easily degraded by human proteases [168]. AMPs have been used as bio-bacteriostatic agents to preserve meat, fruit, juice, soy milk etc., and some of these peptides have been permitted by the US Food and Drug Administration as food additives [169]. AMPs can also reduce lipid oxidation, which leads to the generation of harmful compounds during meat preservation [170]. Active packaging is a promising technique to ensure the quality of food products. Moreover, active packaging is used to carry AMPs in microcapsules and nano-capsules, controlling the release of AMPs to combat microbes during food preservation. However, not all types of AMPs can act as potential ingredients in packaging—this depends on their effectiveness [170,171]. There is a growing demand for food supply as the population increases. AMPs can provide solutions by reducing food waste caused by food spoilage.

Insect AMPs are also applied in agriculture as antibiotic alternatives, avoiding the development of multidrug resistance among microbes [172,173]. For example, insect AMP cecropin AD was supplemented in diets instead of antibiotics, reducing the incidence of diarrhea in piglets due to bacterial infection [174]. Transgenic expression of insect AMPs in plants confers resistance to pathogenic bacteria and fungi [175,176]. In the pharmaceutical industry, AMPs exhibit potential in disease treatment, owing to their ability to selectively target cancer cells and promote cell apoptosis [177,178]. For example, defensins can cooperate with *Drosophila* TNF-like molecule Eiger to drive tumor cell death [179]. Studies show that insect AMPs can inhibit human pathogenic bacteria, making them drug candidates [24]. A nano-delivery system is an effective technology for delivering AMP drugs, but such a system is still in the early stage of development in delivering AMPs; it will be a new research hotspot in the future [180].

## 7. Future Perspectives

Insect AMPs are peptides with heat stability and broad-spectrum bactericidal effects, which make them hotspots for developing insect resources. We summarize the current knowledge of insect AMPs, including classification, distribution patterns in Hexapoda evolution, mechanism of action, and regulation, in this review. However, some underexplored aspects of insect AMPs exist, and we discuss these issues, future perspectives, and challenges for AMP applications in this section.

We summarized the pathways that regulate insect AMP expression, including the Toll, IMD, and JAK-STAT pathways, as well as additional NO, ecdysone, IIS, and JNK signaling. The Toll and IMD pathways are two sole intracellular cascades in Drosophila, and currently, no evidence shows that cross-talk exists between them. Some AMPs are produced only via one signal pathway (e.g., diptericin only for IMD), whereas some are induced from both of the two pathways, such as drosomycin—its systemic expression is induced by the Toll pathway, and local expression is regulated by the IMD pathway in *Drosophila* [107]. Why this certain AMP can be induced by two independent pathways remains to be further explored. Recently, some studies have shown that AMPs can also be induced by nonconventional activation of the Toll pathway in *M. sexta* [4,117]. This reminds us that more possibilities may exist for activating these pathways to produce AMPs among insects. In addition to conserved NF-κB pathways, NO and ecdysone signaling are primarily associated with the IMD pathway to induce the production of AMPs, but how these pathways activate the IMD pathway is currently unknown. So far, studies of the pathways regulating AMP production are primarily focused on the insects of Diptera and Lepidoptera, which are holometabolous insect orders; how most hemimorphic insects (e.g., crickets and locusts) regulate AMP production has been neglected. Similarly, coleopteran insects, which make up more than one-third of insects, also need more attention on their AMP production. This is conducive to the extensive development and utilization of insect AMP resources.

Although certain AMPs exhibit high specificity for a particular pathogen, such as diptericin—which acts specifically on *Providencia rettgeri*—and drosocin defenses against *Enterobacter cloacae* [181,182], we found no single AMP that can be singularly effective against all pathogens (Table 2). This may be the reason why the innate system sustains multiple AMP species. However, several AMPs show similar antimicrobial activities, for instance, attacin, cecropin, defensin, diptericin, and drosocin defense against Gram-negative bacteria in *Drosophila* [114]. It is not an economical strategy for the innate system to maintain the production of functionally overlapping AMPs. Understandably, this strategy prevents pathogens from developing resistance to specific AMPs. This strategy also implies that a synergistic effect may exist between AMPs. While current studies mainly focus on individual AMPs and their production and activity in vitro, more studies need to focus on synergistic effects among AMPs to truly reflect how AMPs work in vivo.

Current studies of the evolution of AMPs are mainly focused on a certain insect order or a specific type of AMPs [64,73]. We provide a more comprehensive study of the evolutionary relationships of AMPs in this review. Understandably, AMPs with the same structure or activity primarily cluster into one group, but that is not the case with some of them (e.g., attacin and gloverin in Figure 1). Further specific sequence and structural analyses are required for explanation of this. The discrepancy may be caused by gene duplication, horizontal gene transfer, and subsequent diversification among these AMPs during the course of evolution. However, we found some insects, such as Protura, Ephemeroptera, Strepasiptera, and some Hemiptera, have no AMP production (Figure 2b). We analyzed and discussed the reasons for the absence of the IMD pathway and AMPs in hemipteran insects based on aphids in terms of fitness costs [85]. Insects may redirect resources allocated to immunity towards various physiological processes, such as nutrition, reproduction, and foraging, due to costly immune responses [183,184]. For instance, adult honeybees prioritize phenoloxidase-based immunity over energetically expensive cellular immunity upon entering the foraging stage [184]. We infer that the lack of AMPs in some species is due to the results of parallel evolution between these insects and adaptation to the unique, developing conditions. Thus, more studies are needed to explore why the insects in Protura, Ephemeroptera, and Strepasiptera do not rely on AMPs to defend against infections.

Besides the applications mentioned above, some insect AMPs exhibit resistance to parasites, and transgenic mosquitoes expressing AMPs have been generated to impede *Plasmodium* and nematode transmission [185,186]. Furthermore, antiviral AMPs are becoming hot research subjects. However, the research on antiviral AMPs is still not in-depth. The following key issues remain for further study: identification, recognition, regulation, and mechanism of action of antiviral AMPs. This research will facilitate the development of antiviral AMPs as antiviral drugs [187]. Although AMPs have gained great attention in industrial applications, some shortcomings limit the wide application of natural AMPs, e.g., poor proteolytic stability, potential hemolysis, high production cost, low bioavailability, and unknown toxicity [82]. Many strategies have been conducted to circumvent these shortcomings by encapsulating and structurally modifying AMPs [82,188]. Although insect cell lines have been proven to be promising systems for producing insect-derived recombinant peptides, the cost of production is still higher than conventional drugs [24]. More approaches are needed in the future to improve biological properties that make insect AMPs favorable in several industries. Faced with numerous insect AMP resources, research on the Black Soldier Fly *Hermetia illucens* (Insecta: Diptera) provides us with new techniques for predicting AMP activity online, which facilitates the screening of promising AMPs for further research in vitro [189]. Due to evolutionary conservatism, the insect immune system has certain similarities with mammals in molecular components and signaling pathways. Insects, especially *Drosophila*, can be used as a powerful genetic tool to explore the diversity functions of AMPs, and they will provide new perspectives for determining the roles of AMPs in complex mammalian systems. In addition to what we have reviewed in this paper, the design, extraction, and production processes of AMPs are also worthy of attention and are conducive to the further application of insect AMP resources in various fields.

## 8. Conclusions

AMPs are not only the effectors in eliminating invading pathogens in insects, but they also show promising applications in the clinical, agriculture, and food industries. In this review, the AMP discoveries, structures, mechanisms of action, antimicrobial activities, and shared characteristics in insects, as well as the types, quantities, and activities of AMPs in each model insect, are summarized; this information provides references for further AMP investigations and applications. Additionally, we conducted the first comprehensive evolutionary analysis of model insect AMPs through phylogenetic analysis, unveiling four distinct groups based on structural and functional similarities. The analysis of AMP gene distribution responsible for encoding these antimicrobial effectors across diverse insect species sheds light on their conservation and uniqueness, which offers a guide for selecting the appropriate types of insects for specific applications. Moreover, the intricate network of classical signaling pathways regulating AMPs, including the classical Toll, IMD, and JAK-STAT pathways, along with additional pathways linked to Nitric Oxide, insulin-like signaling, and insect hormones such as 20E and JH, are reviewed, suggesting the adaptability of insects in responding to diverse environmental challenges. This review not only enhances the comprehension of how AMPs serve as immunity guardians across insect species but also offers insights into using these resources in the food industry.

## Figures and Tables

**Table 1:** Gene names and number of immune-related AMPs from model insects.

AMP Genes	*Dm*	*Ms*	*Bm*	*Ha*	*Tc*	*Ag*	*Am*
*abaecin*	-	-	-	-	-	-	1
*apidaecin*	-	-	-	-	-	-	2
*apisimin*	-	-	-	-	-	-	1
*attacin*	4	11	2	1	3	1	-
*bomanin*	3	-	-	-	-	-	-
*cecropin*	4	15	13	5	3	4	-
*cobatoxin*	-	-	-	1	-	-	-
*coleoptericin*	-	-	-	-	2	-	-
*defensin*	1	6	2	1	4	4	2
*diapausin*	-	14	-	-	-	-	-
*diptericin*	2	-	-	-	-	-	-
*drosocin*	1	-	-	-	-	-	-
*drosomycin*	7	-	-	-	-	-	-
*gambicin*	-	-	-	-	-	-	1
*gloverin*	-	1	4	3	-	-	-
*hymenoptaecin*	-	-	-	-	-	-	1
*lebocin*	-	4	1	1	-	-	-
*metchnikowin*	1	-	-	-	-	-	-
*moricin*	-	6	9	4	-	-	-

*Dm*, *Drosophila melanogaster* (Insecta: Diptera); *Ms*, *Manduca sexta* (Insecta: Lepidoptera), *Bm*, *Bombyx mori* (Insecta: Lepidoptera), *Ha*, *Helicoverpa armigera* (Insecta: Lepidoptera); *Tc*, *Tribolium castaneum* (Insecta: Coleoptera); *Ag*, *Anopheles gambiae* (Insecta: Diptera); *Am*, *Apis mellifera* (Insecta: Hymenoptera). “-”, not found. The counts of AMPs listed in this table are mainly from references [26,27,28,29,30,31,32].

**Table 2:** Model insect antimicrobial peptides and main activity.

AMP Family	Species	Accession Number	Gene Name	Main Activity	Immune Pathway	References
abaecin	*Apis mellifera*	NP_001011617.1	*abaecin*	G^+^, G^−^	Imd	[55]
apidaecin	*Apis mellifera*	NP_001011642.1	*apidaecin I*	G^−^	nd	[61]
apidaecin	*Apis mellifera*	NP_001011613.1	*apidaecin II*	G^−^	nd	[61]
attacin	*Drosophila melanogaster*	NP_523745.1	*attA*	G^−^	Imd	[46]
attacin	*Drosophila melanogaster*	NP_523746.1	*attB*	G^−^	Imd	[46]
attacin	*Drosophila melanogaster*	NP_523729.3	*attC*	G^−^	Imd	[46]
attacin	*Drosophila melanogaster*	NP_524391.2	*attD*	G^−^	Imd	[46,65]
attacin	*Bombyx mori*	ADB08384.1	*attacin*	G^+^, G^−^	nd	[70]
attacin	*Helicoverpa armigera*	ADR51155.1	*att*	G^+^, F	nd	[26]
attacin	*Tribolium castaneum*	XP_001809637.1	*attacin 2*	G^+^, G^−^	nd	[32]
attacin	*Manduca sexta*	AAY82587.1	*attacin-1*	G^+^,	nd	[143]
attacin	*Manduca sexta*	CAL25130.1	*attacin-2*	G^+^, G^−^	nd	[144]
bomanin	*Drosophila melanogaster*	NP_611319.1	*IM1-type*	G^+^, F	Toll	[145]
bomanin	*Drosophila melanogaster*	NP_001262823.1	*CG5778-type*	G^+^, F	Toll	[145]
bomanin	*Drosophila melanogaster*	NP_611318.2	*IM23-type*	G^+^, F	Toll	[145]
cecropin	*Drosophila melanogaster*	NP_524588.1	*cecA1*	G^−^	Imd	[37,45]
cecropin	*Drosophila melanogaster*	NP_524589.1	*cecA2*	G^−^	Imd	[37,45]
cecropin	*Drosophila melanogaster*	NP_524590.1	*cecB*	G^−^	Imd	[37,45]
cecropin	*Drosophila melanogaster*	NP_524591.1	*cecC*	G^−^	Imd	[37,45]
cecropin	*Bombyx mori*	NP_001037462.1	*cec A1*	G^+^, G^−^	nd	[146]
cecropin	*Bombyx mori*	NP_001037460.1	*cecB6*	G^+^, G^−^	nd	[38]
cecropin	*Bombyx mori*	BAL70382.1	*cecD*	G^+^, G^−^	nd	[38]
cecropin	*Bombyx mori*	NP_001037392.1	*cecE*	G^−^	nd	[38]
cecropin	*Helicoverpa armigera*	ADR51154.1	*cecropin-1*	F	nd	[26]
cecropin	*Helicoverpa armigera*	ADR51147.1	*cecropin-2*	G^+^, G^−^	nd	[26]
cecropin	*Helicoverpa armigera*	ADR51148.1	*cecropin-3*	F	nd	[26]
cecropin	*Anopheles gambiae*	AAF22649.1	*cecropin A*	G^+^, G^−^	nd	[147]
cecropin	*Anopheles gambiae*	XP_040173530.1	*cecropin B*	G^+^, G^−^	nd	[147]
cecropin	*Manduca sexta*	AAO74638.1	*cecropin-6*	G^+^	nd	[143]
cobatoxin	*Helicoverpa armigera*	ADR51150.1	*cob*	G^+^, G^−^, F	nd	[26]
defensin	*Drosophila melanogaster*	NP_523672.1	*def*	G^+^, G^−^	Imd	[45]
defensin	*Apis mellifera*	NP_001011616.1	*Royalisin*	G^+^, F	Toll	[148]
defensin	*Bombyx mori*	NP_001037370.1	*def*	G^+^, G^−^, F	Toll, Imd	[49]
defensin	*Tribolium castaneum*	XP_973575.3	*defensin1*	G^+^, G^−^, F	nd	[32]
defensin	*Tribolium castaneum*	XP_968237.2	*defensin2*	G^+^, G^−^, F	nd	[32]
diapausin	*Manduca sexta*	ALP00204.1	*diapausin-1*	F	nd	[149]
diptericin	*Drosophila melanogaster*	NP_476808.1	*dptA*	G^−^	Imd	[45,75]
diptericin	*Drosophila melanogaster*	NP_523787.2	*dptB*	G^−^	Imd	[45,75]
drosocin	*Drosophila melanogaster*	NP_523744.1	*dro*	G^−^	Imd	[58]
drosomycin	*Drosophila melanogaster*	NP_523901.1	*drs*	G^−^, F	Toll, Imd	[50,51]
drosomycin	*Drosophila melanogaster*	NP_728872.1	*drs-like1*	G^−^	JAK-STAT	[45]
drosomycin	*Drosophila melanogaster*	AAF47756.2	*drs-like2*	G^−^	JAK-STAT	[45]
drosomycin	*Drosophila melanogaster*	NP_728861.1	*drs-like3*	G^−^	JAK-STAT	[45]
drosomycin	*Drosophila melanogaster*	NP_728862.1	*drs-like4*	G^−^	JAK-STAT	[45]
drosomycin	*Drosophila melanogaster*	AAF47757.1	*drs-like5*	G^−^	JAK-STAT	[45]
drosomycin	*Drosophila melanogaster*	AAF47765.1	*drs-like6*	G^−^	JAK-STAT	[45]
gambicin	*Anopheles gambiae*	ACA05604.1	*gambicin*	G^+^, G^−^	nd	[150]
gloverin	*Manduca sexta*	CAL25129.1	*glv*	G^+^, G^−^, F	nd	[70]
gloverin	*Bombyx mori*	NP_001036930.1	*glv1*	G^+^, G^−^	nd	[38,75]
gloverin	*Bombyx mori*	NP_001037683.1	*glv2*	G^+^, G^−^	nd	[38,75]
gloverin	*Bombyx mori*	NP_001093312.1	*glv3*	G^+^, G^−^	nd	[38]
gloverin	*Bombyx mori*	NP_001093312.1	*glv4*	G^+^, G^−^	nd	[38]
gloverin	*Helicoverpa armigera*	ADR51146.1	*glo*	G^+^, G^−^, F	nd	[26]
hymenoptaecin	*Apis mellifera*	NP_001011615.1	*hymenoptaecin*	G^+^, G^−^	Imd	[151]
lebocin	*Manduca sexta*	ADE20197.1	*lebocin B*	G^+^, G^−^, F	nd	[55]
lebocin	*Manduca sexta*	XP_030038912.2	*lebocin C*	G^+^, G^−^, F	nd	[55]
lebocin	*Bombyx mori*	sp|P54684.1|	*lebocin 1/2*	G^+^, G^−^	nd	[57]
lebocin	*Bombyx mori*	NP_001119732.2	*lebocin 3*	G^+^, G^−^	nd	[57]
moricin	*Manduca sexta*	sp|Q86MA1.1|	*moricin 1*	G^+^, G^−^	nd	[38,39]
moricin	*Bombyx mori*	NP_001036829.2	*mor*	G^+^, G^−^	nd	[38]
moricin	*Bombyx mori*	pdb|1KV4|	*morLA1*	G^+^, G^−^	nd	[38]
moricin	*Helicoverpa armigera*	ADR51149.1	*mor*	G^+^, G^−^, F	nd	[26]
metchnkowin	*Drosophila melanogaster*	NP_523752.1	*mtk*	G^+^, G^−^, F	Toll, Imd	[45,152]

G^+^, Gram-positive bacteria; G^−^, Gram-negative bacteria; F, Fungi; nd, not determined.

## Data Availability

All data described in this manuscript are entirely available in this article.

## Supplementary Materials

The following supporting information can be downloaded at: https://www.mdpi.com/article/10.3390/ijms25073835/s1. References [190,191,192,193,194,195,196,197,198,199,200] are cited in the Supplementary Materials.
