# Supplementary material for: Insect Antimicrobial Peptides as Guardians of Immunity and Beyond: A Review
Source: Int J Mol Sci. 2024 Mar 29;25(7):3835. doi: 10.3390/ijms25073835 (PMC11011964; doi:10.3390/ijms25073835)
Supplement: Supplementary file 1 [file ijms-25-03835-s001.zip › ijms-2857496-supplementary.pdf]

Supplementary file S1

Molecular methods

Phylogeny of AMPs

A total of 247 AMP protein sequences were retrieved from publications and from NCBI (**Table S1**), which were then aligned with the mafft-linsi algorithm of MAFFT (v7.487) [190]. The alignment was then checked by eyes using AliView (v1.28) [191]. A Maximum-likelihood (ML) tree of AMP was constructed with IQ-TREE (v2.2.0) [192]. The best-fitting substitution models were automatically determined by the ModelFinder algorithm [193] in IQ-TREE. In total, 40 independent IQ-TREE runs (--runs 40), a smaller perturbation strength (-pers 0.2) and larger number of stop iterations (-nstop 500) were applied to avoid local optima during heuristics. SH-aLRT branch test [194] was performed using 2000 bootstrap replicates (-alrt 2000). Tree visualizations were finished with the Newick utilities (version 1.6) [195] and ggtree (v 3.8.2) [196].

Identification of AMPs in insects

We further identified AMPs of insects based on the transcriptome dataset from Misof et al. 2014 [84] and genome dataset from NCBI using profile hidden Markov models (pHMM) [197,198] (**Table S2**). In brief, the HMM profile of each AMP gene was constructed based on the AMP alignment with the hmmbuild command in HMMER (v 3.1b2) (http://hmmer.org/). For the genome dataset, the protein set of each genome was directly downloaded from NCBI. For the transcriptome dataset, each sequence was translated into six frames using seqkit (v0.13.2) [199]. The initial AMP sequences were screened out with the hmmscan command of HMMER (v 3.1b2), with e-value cutoff 10^{-3}. To further filter out the potential false-positive hits, which could be due to the relative short length of AMP, we performed blastp (v2.9.0) [200] search against the above mentioned 247 AMP protein sequences with a e-value cutoff 10^{-5}. The gene assignment of each sequence was based on its best-hit.
